# The biology of extracellular vesicles: The known unknowns

**DOI:** 10.1371/journal.pbio.3000363

**Published:** 2019-07-18

**Authors:** Leonid Margolis, Yoel Sadovsky

**Affiliations:** 1 Section for Intercellular Interactions, Eunice Kennedy-Shriver National Institute of Child Health and Human Development, NIH, Bethesda, Maryland, United States of America; 2 Magee-Womens Research Institute, University of Pittsburgh, Pittsburgh, Pennsylvania, United States of America; 3 Department of Obstetrics, Gynecology and Reproductive Sciences, University of Pittsburgh, Pittsburgh, Pennsylvania, United States of America; 4 Department of Microbiology and Molecular Genetics, University of Pittsburgh, Pittsburgh, Pennsylvania, United States of America

## Abstract

For many years, double-layer phospholipid membrane vesicles, released by most cells, were not considered to be of biological significance. This stance has dramatically changed with the recognition of extracellular vesicles (EVs) as carriers of biologically active molecules that can traffic to local or distant targets and execute defined biological functions. The dimensionality of the field has expanded with the appreciation of diverse types of EVs and the complexity of vesicle biogenesis, cargo loading, release pathways, targeting mechanisms, and vesicle processing. With the expanded interest in the field and the accelerated rate of publications on EV structure and function in diverse biomedical fields, it has become difficult to distinguish between well-established biological features of EV and the untested hypotheses or speculative assumptions that await experimental proof. With the growing interest despite the limited evidence, we sought in this essay to formulate a set of unsolved mysteries in the field, sort out established data from fascinating hypotheses, and formulate several challenging questions that must be answered for the field to advance.

## Abstract 2

As the field of extracellular vesicles rapidly expands, many questions remain unanswered; this Unsolved Mystery article provides a systematic compilation of these challenges, and formulates a path to addressing them.

## Introduction: Primitive vesicles go complex

During the course of evolution, the creation of the first cell required sequestration of encoding nucleic acids and their replication machinery from the hazardous external microenvironment. Lipids with polar head groups and hydrophobic tails became a natural solution to this challenge. In the case of phospholipids, for example, a layer of hydrophilic polar head groups and two hydrophobic lipid tails readily form double-layer membranes in the aqueous solution. Moreover, in light of the hydrophobic nature of the tails, the phospholipid membrane required a configuration that minimized edges, resulting in a sphere. The formation of spherical double-layer phospholipid structures in aqueous media occurs spontaneously without enzymatic activity.

Phospholipid vesicles are probably the simplest biological structures whose biogenesis can be easily reproduced in a tube. It is therefore not surprising that double-layer phospholipid membranes were used to encapsulate biological molecules. In addition, phospholipid membranes acquired new functions during evolution. For example, phospholipid membranes of enveloped viruses incorporated proteins that mediate viral recognition and entry into target cells. The phospholipid membrane that surrounds cells is even more complex and contains proteins that regulate inflow and outflow of small molecules, transmit electrical currents, and modulate ligand–receptor interactions as a part of cellular communication.

For many years, scientists seeking to understand the function of these complex phospholipid membranes studied them in cells or viruses but largely ignored the more primitive phospholipid vesicles. Indeed, their apparent simplicity, minute size, and unknown function kept released subcellular vesicles under the research radar screen. They have been regarded as cellular debris and, reflecting their high production by thrombocytes, were dubbed “platelet dust.” This sentiment has recently changed with the advent of new data on exocytosis; improved tools for vesicle isolation, measurement, and imaging; and most importantly, experimentally supported functions alongside creative hypotheses regarding their action. Released vesicles, now clustered under the term “extracellular vesicles” (EVs) [1], became a commonplace object of investigation in literally every field of biomedicine. Indeed, EVs are ubiquitous. They are released by bacteria [2] and by virtually every cell in multicellular organisms. They entrap nucleic acids, diverse cellular proteins, and metabolites and are predicted to transfer their packaged molecules from one cell to another; therefore, they are dubbed “Trojan horses” in the pioneering text by Gould and Hildreth [3].

Although the field of EV research is new, “there is no new thing under the sun” [4]. Indeed, we can trace the concept of EVs as far as Charles Darwin, who 150 years ago proposed (as a part of his pangenesis theory) that every cell type in the body generates gemmules, which are particles of “minute size” that contain molecules and serve to communicate them to other cell types. Gemmules may also mediate the maternal–fetal transfer of heritable information and may be subject to environmental modification. At that time, Darwin’s theory was not accepted and went into hibernation because of lack of evidence. However, contemporary readers can easily relate Darwin’s gemmules to EVs that, although probably not inherited, nevertheless carry nucleic acids [5] and mediate mother-to-fetus dialog [6].

**Fig 1:**
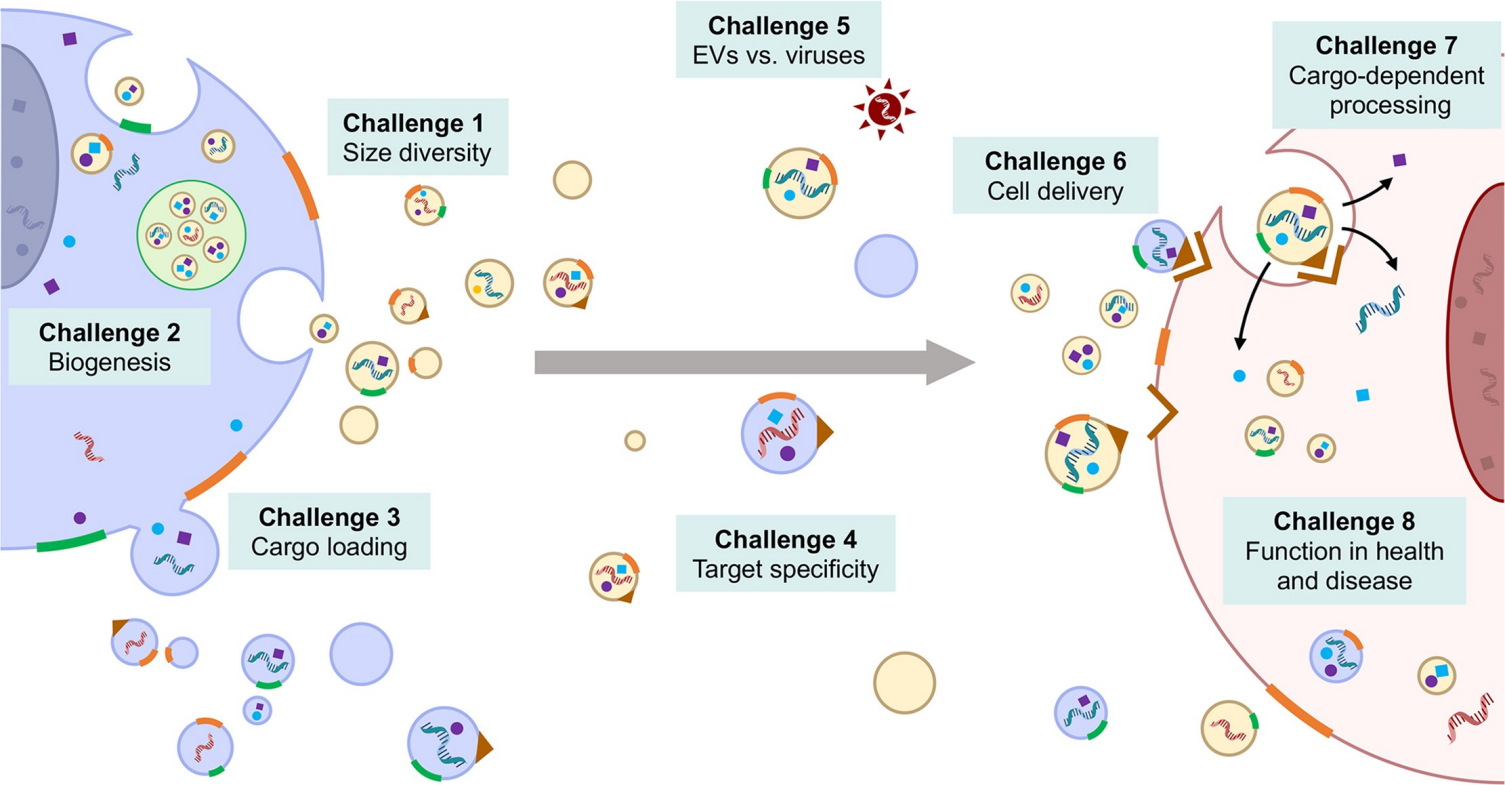
Progress in the field of EV biology depends on solving at least eight challenging problems, which refer to the “unsolved mysteries” described in the corresponding text sections. EV, extracellular vesicle.

The explosion of new data about EVs, which paved the way to a new field of research, was eclipsed by an even grander explosion of untested hypotheses by overly enthusiastic researchers outside of the EV field. The use of shared terminologies for structurally and functionally diverse vesicles attests to the inadequate EV separation and analysis techniques. EVs released by cells are highly heterogeneous in size, cargo, membrane composition, biogenesis, and most importantly, biological function. Although the recent communications and guidelines by the Extracellular RNA Communication Consortium (ERCC1) and the International Society for Extracellular Vesicles (ISEV) clearly assist in EV definitions, isolation methods, and information on EV profiles in diverse biofluids [7–9], a lack of standards remains widespread. Many vital aspects of EV function, including the selective incorporation of cellular molecules, release of EVs to the extracellular space, recognition and acceptance by other cells, and high efficiency of delivery of bioactive molecules to the recipient cells, remain a mystery. These are particularly enigmatic when the function of EVs in their natural environment, not merely in simulated biological models, is assessed. Below, we discuss the main unsolved mysteries and technical hurdles that challenge the field of EV research and propose priorities and clues for solving these mysteries. These challenges are also depicted in Fig 1.

## Size matters: EV subtypes

Within any field of biology, definitions and classifications naturally lag behind essential knowledge, obtained through experiments and the accrual of scientific observations, of the structures and functions involved. At present, common definitions of EVs rely on size. In view of the range of phospholipid membrane thickness, the smallest diameter of phospholipid vesicles may be in the range of 10–20 nm [10]. The diameter of most EVs is likely near 100 nm and above. The upper size limit of EVs is not known. The size of apoptotic bodies and “oncosomes” [11] is in the range of microns. Strictly speaking, mammalian platelets, which are approximately 2 μm in diameter, can be considered megakaryocyte-generated EVs. Practically, most studies are focused on EVs that measure 1 μm or less.

The recent deployment of asymmetric flow field-flow fractionation has revealed distinct populations of larger and smaller EVs, including the 35-nm-in-diameter exomeres [12]. Other nanoparticles that may or may not be EVs were also revealed using PKH lipophilic dye staining of precipitated and filtered particles [13]. Energetic and thermodynamic analyses of membrane bending, adjusted to membrane thickness and elasticity, clearly predict that spontaneous vesiculation will result in a range of EV configurations and size distribution. Yet actual vesicle size depends not only on the type of membrane phospholipids but also on the presence or absence of particular membrane proteins. Currently, a popular way to define the distribution of EV subtypes is by using nanoparticle tracking analysis (NTA) systems. Although easy to use, the results of such evaluation may be misleading, as NTA or similar tools analyzes all particles merely on the basis of size. The data generated by NTA systems should be verified by electron microscopy (EM)—in particular, by cryo-EM, flow cytometry, and atomic force microscopy [14]. A fine EV isolation and size analysis based on step-gradient centrifugation seems to be adequate, as reported by Théry and colleagues [9,15]. Further analysis of EV subpopulations will benefit from linking size to other biophysical and biochemical EV properties in order to better define vesicle subtype, as discussed in the next section.

### Unsolved mystery #1

What are the biological implications of the broad EV size diversity? If every size-defined EV has different functions, the system of EV-mediated cell–cell communication is much more complex than currently assumed. To address this issue, we need to identify the smallest size difference that faithfully defines different vesicles. Can one cell produce EVs of different sizes, or does size difference reflect EV production by different cells? Does the repertoire of EVs released by a particular cell type change over time? What additional variables should be included to better define EV homogeneity and heterogeneity?

## Beyond size: Composition matters as well

At first glance, the ability of a single cell type to release myriad EVs of different sizes seemed enigmatic. It is now clear that there are at least three distinct EV biogenetic pathways: endosomal sorting complexes required for transport (ESCRT) protein–based formation of intraluminal vesicles within multivesicular bodies (MVBs) (“exosomes”), a pathway that is shared by viruses [16]; formation by pinching off from the plasma membrane (“microvesicles” [17,18]); and membrane disintegration (“apoptotic bodies”). Whether vesicle classification by biogenesis pathway rather than by size may have a stronger biological basis or functional relevance remains an unanswered question. Are there sets of EV molecules that are specific to a particular biogenesis pathway and that would allow us to distinguish different EVs? For example, Ras-associated binding (Rab) proteins Rab27a and Rab27b, both important for microvesicle docking [19], may help to distinguish exosomes from microvesicles. Another strategy that may help in distinguishing between exosomes and the smaller microvesicles is to isolate MVBs and extract their intraluminal exosomes before they are released. However, isolation of MVBs remains a challenge.

### Unsolved mystery #2

What is the contribution of biogenesis pathway and composition to the definition of EV biology? The enormous complexity of EV biology can be somewhat reduced by linking structural EV variables with function. The relevant variables remain to be defined and likely include more than merely size and composition. More biologically insightful links between EV size and structure, including membrane molecules and cargo, may assist in advancing our definitions of EVs.

## Preparation of a parcel: Is the cargo sorted by package type?

During their biogenesis, EVs may selectively capture cell-specific proteins, lipids, RNAs, or even DNA, which may become a part of the EV membrane’s or cargo’s “molecular signature” (reviewed in [20]). Based on these discriminatory signatures, particular EVs, isolated from complex mixtures (e.g., blood), may be traced to their cells of origin. However, the mechanism of such selective packaging remains unknown. Selectivity of cargo packaging into EVs is best demonstrated by RNA molecules, as the spectra of EV RNA usually reflect the RNA spectra of the donor cells [21]. Several hnRNPq RNA binding proteins (RBPs, such as YBX1, KRAS, hnRNPA2B1, SYNCRIP, HuR, and ELAVL1 [22–26]) have been implicated in the sorting of RNAs into EVs. Proteins may also be selectively sorted to EVs: for example, cytokines associated with EVs are different from those released in a soluble form by cells of the same type [27]. However, the selectivity of EV encapsulation may not be a universal rule, as other data suggest a nonselective micro RNA (miRNA) packaging and secretion [28,29].

Encapsulation of cargo within EVs may be enough to protect it against degradation; however, additional RNA protection by intravesicular RBPs was recently suggested [30]. The presence of RNA-processing enzymes, such as DICER, within EVs [31] also suggests the tantalizing possibility that the packages are not inert and that there is ongoing intravesicular processing of cargo molecules. Notably, the previous finding of AGO2 within EV cargo rather than in ribonucleoprotein complexes has been recently disputed [32].

Different molecules may be concomitantly incorporated into EVs. In general, biological communication systems are characterized by redundancies and interdependence, such that one variable is associated with other variables. For instance, when we receive an envelope with an Internal Revenue Service logo, we reasonably expect that there is no love letter inside. If EVs indeed constitute a system of cell–cell communication, they are likely to harbor some redundancy in surface characteristics and cargo, as recently reported [33].

### Unsolved mystery #3

Can we identify redundancies within EV cargo and relations among EV cargo elements? Addressing this issue may require the development of an elaborate single-vesicle analysis tool rather than a bulk analysis of the entire EV population. We should decipher the mechanism of selective encapsulation of particular cargo molecules into EVs and its association with EV membrane molecules.

## You’ve got mail

The quantity of cargo material carried within an EV is extremely small. For example, if one calculates the total amount of miRNA or other molecules that they carry, it is a mystery how EVs can execute their assumed functions [34]. Thus, for a relatively small number of EVs to carry particular bioactive molecules into discrete cell populations, one has to assume a highly efficient and EV-specific recognition tool in recipient cells [18]. Whereas distinct targeting may be superfluous when a large number of EVs or bioactive molecules are released to neighboring cells, delivery to a distant target depends on a unique address or “bar code” provided by a combination of specific surface molecules that can be recognized by a “bar code reader” (receptor molecules) on the target cell’s surface. The nature of this bar code, which likely consists of proteins and lipids on the EV surface, remains a central question in the field [35,36]. It is possible that certain cells attract circulating EVs, whereas others reject them, passively or actively, by altering EV adhesion to the cell surface and, thus, subsequent internalization.

Cytokines found on the EV surface may serve as one type of bar code molecule and may be recognized by the abundant cell-specific cytokine receptors [27]. Cytokine association with EVs was reported to change in HIV infection, but their impact on EV interaction remains unclear [14].

Although the concept of EV delivery of bioactive molecules in order to impact target cell physiology is attractive, a simpler model may suggest that EVs are a means to purge cellular cargo and thus avoid cell-autonomous effects. This has been recently suggested with regard to the high prevalence of tRNAs and vault RNAs within EVs [30,37] but has not been fully assessed.

### Unsolved mystery #4

Are the nature and quantity of cargo material sufficient to explain the ascribed biological effects? As a first approach, we should calculate more precisely how many bioactive molecules EVs can deliver to the target cells. A central challenge is to determine the “bar code” that destines particular EVs to a particular cell and the target cell bar code reader that responds to EV targeting. Solving these questions may pave the way to tools designed to manipulate the bar code reader, which has important translational implications.

## Exosomes going viral

Exosomes and many types of enveloped viral particles, particularly RNA viruses, have a similar size and are generated through the same ESCRT pathways (reviewed in [18]), making exosomes and RNA viruses “close relatives” [38]. In fact, enveloped viruses may be considered a form of EV. Exosomes from virus-infected cells incorporate not only cell-encoded but also virus-encoded molecules. For example, EVs generated by cells infected with HIV or cytomegalovirus (CMV) carry virus-encoded membrane proteins that are involved in virus recognition during cell entry [39,40]. Similarly, the biogenesis pathways of RNA virions (e.g., HIV) and EVs result in formation of particles with similar physicochemical properties. Moreover, exosomes and small enveloped viruses may have common evolutionary roots. However, we do not know which was the “chicken” and which the “egg”; either enveloped viruses developed from primitive lipid vesicles by encapsulating nucleic acids and incorporating specific membrane molecules for which cells have receptors, or EVs evolved as defective viruses that lost the machinery for nucleic acid replication and membrane molecules that mediate viral infection. New insights into the coevolution of viruses and EVs may shed light on their cross talk and the function of EVs in attenuating viral infections. Interestingly, EVs that are generated by HIV-1-infected cells and harbor viral proteins and viral genome fragments are not infectious, but they can affect viral infectivity itself [41]. EVs may help viruses to evade the immune system [42,43] and promote HIV infection [44]. EVs containing hepatitis C virus (HCV) RNA can transfer HCV infection of cultured hepatocytes [45]. It has also been reported that large EVs can encapsulate the entire hepatitis A virus [46], providing an envelope to this unenveloped virus.

### Unsolved mystery #5

Beyond semantics, what are the differences, in terms of physicochemical properties, between EVs that carry virally encoded molecules and EVs that carry (defective) viral particles? To address this mystery, we should learn how to separate infectious HIV-1 virions (and other small RNA viruses) from the noninfectious EVs carrying viral components. This will allow us to investigate how noninfectious EVs affect viral infection and to try to manipulate EVs to inhibit viral infection.

## A parcel is delivered: How to open it?

Specific mechanisms are needed to deliver bioactive EV cargo inside the cell. The processing of the EV message, embedded within its bioactive material, remains an unsolved mystery in the field. Current data suggest that different cell types use specific pathways to promote EV entry into cells for subsequent intracellular processing [18,36,47]. This may involve discrete endocytic pathways, fusion of EVs with the plasma membrane, or other, yet-to-be-identified strategies. Selective uptake may also be intertwined with the means used for cell-dependent EV recognition. The role of EV cargo in this process is unknown.

In principle, cell entry may not be obligatory for EV function. Binding to particular cell-surface molecules may trigger a cascade of reactions that alter cell physiology. Such a phenomenon is known for viruses (“kiss and run”) [48] or for peptide complexes on the EV surface that interact with T-cell receptors on T lymphocytes [49]. Also, EVs may collapse on the cell surface, releasing a protein cargo that interacts with specific growth factors, cytokines, or other cell-surface receptors. At the present time, we do not fully understand the diverse mechanisms that mediate the delivery of EV cargo molecules to cell-surface receptors and into the cells. To ensure more definitive and reproducible results, the delineation of pathways using pharmacological inhibitors, which may have nonspecific effects, is not sufficient. Multiple genomic knock-down or knock-out tools should accompany such inhibitors to decipher EV release and uptake mechanisms, and discrepancies among these approaches should be discussed [50].

### Unsolved mystery #6

What are the mechanisms underlying the efficient unpacking of EVs in target cells? We need to decipher the mechanisms underlying EV entry into and cargo processing inside the cells. Can EVs affect the cells by merely binding to the plasma membrane? How can EV cargo molecules evade degradation in the target cell’s cytoplasm?

## Executing the received order

Although it is generally believed that EVs generated by one type of cell can influence the function of target cells, there are only a limited number of well-substantiated examples of vesicles generated by one cell type impacting the biology of a different cell or tissue type [51–55], with most such data derived from in vitro systems. It might be easier to imagine how a replication-competent RNA molecule may affect target cell physiology [37]. A similar expectation from a small amount of protein is more challenging and may involve amplification of the cargo protein’s signals.

EVs carrying “executive orders” in the body can emanate not only from the host cells but also from microbiota. Recently, it was established that bacteria, including commensal ones, release EVs (reviewed in [2]), and the spectra of these EVs change in pathologies [56], stimulating the tantalizing hypothesis that bacterial EVs mediate microbiota–host interactions.

### Unsolved mystery #7

How do EVs alter cell physiology? Many systems have been proposed, yet definitive proofs, particularly using in vivo systems, are scarce.

## Healthy versus diseased packages

Several diseases have been recently associated with abnormalities in EV-mediated cell–cell communications. In this context, a disease may emanate from EV miscommunication at the level of the sending cells, the target cells, or the circulating EVs themselves and may involve the number of packages, their composition, delivery, or processing systems. Examples of studies in which EVs have been implicated in disease processes include cancer and metastatic spread [51, 57–59], cardiometabolic disease and regulation of myocardial hypertrophy [60], cardiac regeneration after injury [61,62], obesity [63], and neurological disease [64,65]. EVs were also implicated in defense against a disease (e.g., antiviral effect [54]). Is it also possible that global changes in the vesiculome (akin to changes in the microbiome) may affect general homeostatic functions throughout the entire organism? The link between EVs and diseases led researchers to examine EV pathways as targets for therapeutic intervention, as well as the isolation and analysis of tissue-specific circulating EVs as biomarkers of diseases (reviewed by [62]). Indeed, blood EVs are particularly attractive as “liquid biopsies” for determining the state of hard-to-access tissues. The utilization of EVs as diagnostic tools requires improved and standardized EV isolation technologies that may exceed current standards (e.g., ultracentrifugation/gradient-based separation, antibody-based chromatography columns) and may also require faster, near-point-of-care isolation [15,66]. Recently, De Wever and colleageus summarized different issues that should be resolved in order to promote the clinical applications of EVs [67]. Whether or not altered EVs play a functional role in pathogenesis or are merely a symptom may be resolved with future EV manipulation or the delivery of artificial EVs to minimize disease.

### Unsolved mystery #8

Do disease-associated EVs play a role in disease pathogenesis, or are they merely a marker of disease? If EVs are a part of disease development or severity, their manipulation could have important therapeutic implications. EVs may also serve an important role in tissue-specific clinical diagnostics.

## Concluding remarks

The field of EV biology is rapidly evolving and expanding, affecting almost all biomedical disciplines, from oncology and obstetrics to microbiology and marine biology. A major challenge in EV research is the huge and underappreciated vesicle diversity. Many of the hurdles in understanding EV function stem from our inability to separate a complex population of vesicles into subclasses of particular sizes, compositions, and biogenetic pathways. Despite a burst of new publications on EVs, several basic hypotheses regarding their function remain to be experimentally tested. Notably, most studies in the field of EV biology have been performed in vitro and using cell lines in which the experiment-specifc culture conditions may affect the biochemical and biophysical features of EVs. Which of these experimental results can be extrapolated to an in vivo system? Notwithstanding occasional skepticism, at least a couple of observations have been proven beyond doubt, justifying the existence of the field: cells release EVs not only in vitro but also in vivo, and diverse types of vesicles have been isolated and analyzed from all bodily fluids. Moreover, the number of EVs and their composition changes in disease provide hope that EV-based liquid biopsies can be used for diagnostics [8,68].

EV-based cell–cell communication relies on the ability of vesicles to deliver bioactive molecules to other cells [69]. This has been validated predominantly in vitro. Recent experiments using animal models [70–72] enable the tracing of EVs to their cells of origin, providing strong evidence in support of an important EV function in vivo.

Data reproducibility remains a challenge in the field. This challenge is amplified by the EV diversity in cultured cells and in bodily fluids. Small deviations in the isolation protocols may result in a collection of different EV populations. The problem of irreproducibility among labs may be solved by the development and deployment of effective technologies that allow better EV isolation, size characterization, and definition of cargo composition. The position papers by ISEV and by ERCC [7–9] and the web platforms and information by the Transparent Reporting and Centralizing Knowledge in Extracellular Vesicle Research (EV-TRACK) consortium ([73], www.evtrack.org) represent important steps in overcoming these problems. More needs to be done in the biomedical research space.

As with other scientific disciplines, EV science is moving forward by a combination of new ideas, technologies, astute observations, and careful data analysis. Currently, the EV field seems to be rich in ideas but lacking in appropriate technologies to test these ideas. Progress is made and is likely to help resolve some of the mysteries listed here. One example is in technologies that target single-vesicle analysis [74,75], which will provide greater insights into vesicle type and diversity. The development of new technologies and their acceptance by scientists in different research fields will reveal new functional and structural properties of EVs that will eventually propel the field to the forefront of biomedicine, on a par, eventually, with other general fields, such as virology, cell biology, and immunology.

## Abbreviations

CMV: cytomegalovirus
EM: electron microscopy
ERCCI: Extracellular RNA Communication Consortium
ESCRT: endosomal sorting complexes required for transport
EV: extracellular vesicle
EV-TRACK: Transparent Reporting and Centralizing Knowledge in Extracellular Vesicle Research
HCV: hepatitis C virus
ISEV: International Society for Extracellular Vesicles
miRNA: micro RNA
MVB: multivesicular body
NTA: nanoparticle tracking analysis
Rab: Ras-associated binding
RBP: RNA binding protein
